# Insulin resistance mediates obesity-related risk of cardiovascular disease: a prospective cohort study

**DOI:** 10.1186/s12933-022-01729-9

**Published:** 2022-12-23

**Authors:** Xue Tian, Shuohua Chen, Penglian Wang, Qin Xu, Yijun Zhang, Yanxia Luo, Shouling Wu, Anxin Wang

**Affiliations:** 1 Department of Neurology, Beijing Tiantan Hospital, Capital Medical University, Beijing, China; 2 Beijing Tiantan Hospital, China National Clinical Research Center for Neurological Diseases, Capital Medical University, No.119 South 4th Ring West Road, Fengtai District, Beijing, 100070 China; 3 Department of Epidemiology and Health Statistics, School of Public Health, Capital Medical University, Beijing, China; 4 Beijing Municipal Key Laboratory of Clinical Epidemiology, Beijing, China; 5 Department of Cardiology, Kailuan Hospital, North China University of Science and Technology, 57 Xinhua East Road, Tangshan, 063000 China

**Keywords:** General obesity, Central obesity, Insulin resistance, The triglyceride-glucose index, Cardiovascular disease, Mediation analysis

## Abstract

**Background:**

The mechanisms linking obesity to cardiovascular disease (CVD) are still not clearly defined. Individuals who are overweight or obese often develop insulin resistance, mediation of the association between obesity and CVD through the insulin resistance seems plausible and has not been investigated. This study aimed to evaluate whether and to what extend the effect of general and central obesity on cardiovascular disease (CVD) is mediated by insulin resistance.

**Methods:**

A total of 94,136 participants without CVD at baseline were recruited from the Kailuan study. Insulin resistance was evaluated by the triglyceride-glucose (TyG) index, calculating as ln [fasting triglyceride (mg/dL) × fasting glucose (mg/dL)/2]. Mediation analysis using a new 2-stage regression method for survival data proposed by Valeri and VanderWeele was to explore the mediating effects of the TyG index on the association between obesity and CVD.

**Results:**

During a median follow-up of 13.01 years, we identified 7327 cases of CVD. Mediation analyses showed that 47.81% of the total association (hazard ratio [HR], 1.18; 95% confidence interval [CI], 1.12–1.24) between overweight and CVD was mediated through the TyG index (HR [indirect association], 1.07; 95% CI, 1.07–1.09), and the proportion mediated was 37.94% for general obesity. For central obesity, analysis by waist circumference, waist/hip, and waist/height categories yielded an attenuated proportion mediated of 32.01, 35.02, and 31.06% for obesity, taken normal weight as reference.

**Conclusions:**

The association between obesity and CVD was mediated by TyG index, suggesting proper control of insulin resistance can be effective to reduce the effects of obesity on CVD.

**Supplementary Information:**

The online version contains supplementary material available at 10.1186/s12933-022-01729-9.

## Background

The obesity epidemic is an emerging public health problem with substantial consequences for health care expenditure and overall quality of life and wellbeing [1]. The increase in obesity prevalence is reported worldwide and is expected to reach 57.8% by 2030 [2]. A large body of data have underscored the deleterious effect of obesity on the risk of cardiovascular disease (CVD) [3–5], which is the leading cause of death and disability worldwide [6]. The increasing health and economic burden of obesity and the failure of implemented interventions to control this health problem have led researchers to identify other causal pathways between obesity and CVD.

Although multiple neurohumoral, metabolic, and hemodynamic components have been suggested as factors in an association between obesity and CVD, the exact mechanisms are still not fully understood [3–5]. Decreased insulin sensitivity might be one component, it has been reported that inflammation and oxidative stress induced by obesity is linked to the development of local and systemic insulin resistance [7]. On the other hand, insulin resistance can lead to endothelial dysfunction, and is contributed to the formation of atherosclerotic plaques by changing the gene expression pattern associated with estrogen receptor, thus may play a critical role in the pathology of CVD [8, 9]. This arise the hypothesis that insulin resistance may provide an indirect pathway by which obesity may affect CVD. The gold standard to define insulin resistance is hyperinsulinemic-euglycemic clamp, but this technique is time-consuming, costly, and complex thus is difficult to use in large epidemiology investigations [10–12]. In contract, The triglyceride-glucose (TyG) index, which is derived from fasting triglyceride (TG) and fasting blood glucose (FBG), has been proposed as a reliable and simple marker of insulin resistance [13, 14].

Therefore, in this study, we aimed to assess whether and to what extend the association of general and central obesity with CVD is mediated through the TyG index, using data from a large community-based prospective cohort study.

## Material and methods

### Study population

Data were deprived from the Kailuan study, which is a prospective cohort study conducted in the Kailuan community in Tangshan, China. The detailed study design and procedures have been published previously [15–17]. Briefly, during June 2006 to October 2007, a total of 101,510 participants (81,110 men and 20,400 women, aged 18–98 years) were enrolled in the baseline survey and have completed questionnaires and health assessments biennially since 2006. All the participants were followed up until their death or December 31, 2019. Participants were excluded if they had a history of stroke or myocardial infarction (MI) (3669), or had missing data on anthropometric parameters, FBG, or TG (3705) at baseline. Ultimately, we included 94,136 participants in the current analysis (Additional file 1: Figure S1). The study was performed according to the guidelines of the Helsinki Declaration and was approved by the Ethics Committee of Kailuan General Hospital and Beijing Tiantan Hospital. All participants provided written informed consent.

### Data collection

Demographic data, including age, sex, education, and income, as well as lifestyle behaviors, such as smoking status, drinking status, and medical history were collected via standardized questionnaires. Educational attainment was categorized as illiterate or primary school, middle school, or high school or above. The family income was categorized as < 800 and ≥ 800 yuan/month. Smoking and drinking status were classified as never, former or current, according to self-reported information. Blood pressure was measured in the in the seated position using a mercury sphygmomanometer, the average of 3 readings were calculated as systolic blood pressure (SBP) and diastolic blood pressure (DBP).

Anthropometric parameters were measured by trained filed workers. WC was measured in centimeters with non-stretchable tape held at the level of the naval while the subject was standing without clothing. Hip circumference was measured around the widest portion of the buttocks with the tape parallel to the floor. Height was measured to the nearest 0.1 cm using a tape rule, and weight was measured to the nearest 0.1 kg using calibrated platform scales.

Blood sample collections were conducted following an 8- to 12-h overnight fast. FBG was measured with the hexokinase/glucose-6-phosphate dehydrogenase method. The CV using blind quality control specimens was < 2.0%. Total cholesterol (TC), TG, high density lipoprotein cholesterol (HDL-C), low density lipoprotein cholesterol (LDL-C), creatinine, and high-sensitivity C-reactive protein (hs-CRP) were assessed by an auto-analyzer (Hitachi 747; Hitachi) at the central laboratory of Kailuan hospital. Hypertension was defined as SBP ≥ 140 mm Hg or DBP ≥ 90 mm Hg, any use of the antihypertensive drug, or self-reported history of hypertension. Diabetes was defined as FBG ≥ 7.0 mmol/L, any use of glucose-lowing drugs, or any self-reported history of diabetes. Dyslipidemia was defined as any self-reported history or use of lipid-lowering drugs, or TC ≥ 6.2 mmol/L, TG ≥ 2.3 mmol/L, LDL-C ≥ 4.1 mmol/L, or HDL-C < 1.0 mmol/L.

### Calculation of obesity indexes and the TyG index

Obesity indexes in our study include body mass index (BMI), waist circumference (WC), waist-to-hip ratio (WHR), and waist-height ratio (WHTR). BMI was calculated as weight in kilograms dividing by the square of height in meter. WHR and WHTR were calculated as WC/hip and WC/height, respectively. According to the Guidelines and Prevention and Control of Overweight and Obesity in Chinese Adults [18], general obesity was defined as a four-category variable: BMI < 18.5 kg/m^2^ for underweight, 18.5 ≤ BMI < 25 km/m^2^ for normal weight, 25 ≤ BMI < 28 km/m^2^ for overweight and BMI ≥ 28 kg/m^2^ for obesity. Central obesity was defined as: (1) WC ≥ 90 cm in men and ≥ 85 cm in women; (2) WHR ≥ 0.9 in men or ≥ 0.8 in women; and (3) WHTR ≥ 0.6. The TyG index was calculated as ln (fasting TG [mg/dl] × FBG [mg/dl]/2) [19, 20].

### Assessment of CVD

Participants were followed up via face-to-face interviews at every 2-year routine medical examination until event of interest, December 31, 2019, or death. The outcomes in the present study were the first occurrence of CVD, including fatal and non-fatal stroke and MI. The database of CVD diagnoses was obtained from the Municipal Social Insurance Institution and Hospital Discharge Register and was updated annually during the follow-up period. An expert panel collected and reviewed annual discharge records from 11 local hospitals to identify patients who were suspected of CVD. Ascertainment of incident stroke and MI was described previously [16, 19–21]. Stroke was diagnosis based on neurological signs, clinical symptoms, and neuroimaging tests, including computed tomography or magnetic resonance, according to the World Health Organization criteria [22]. MI was diagnosed according to the criteria of the World Health Organization on the basis of clinical symptoms, changes in the serum concentrations of cardiac enzymes and biomarkers, and electrocardiographic results [23].

### Statistical analysis

Baseline characteristics are presented as the mean ± standard deviation or percentage. Differences between incident CVD and non-CVD participants were compared using Student’s t test or Wilcoxon for continuous variables and chi-square test for categorical variables. Linear regression was used to assess the association between four obesity indexes and the TyG index.

Mediation analysis for the association of general and central obesity (exposure) with CVD (outcome) through the TyG index (mediator) was evaluated by the 2-stage regression method for survival data proposed by VanderWeele [24]. In brief, 2 regression models are for to the data, one modeling the mediator and the other modeling the outcome; parameters estimates and standard errors of these 2 separate models are combined according to the formulas given therein to obtain estimates for effect size of mediation. We modeled the outcome (CVD) using Cox proportional hazards regression models, and the mediator (the TyG index) using linear regression. All models were adjusted for age, sex, education, income, smoking status, drinking status, history of hypertension, diabetes, dyslipidemia, antihypertensive agents, antidiabetic agents, lipide-lowering agents, SBP, DBP, TC, HDL-C, and high sensitivity C-reactive protein, without inclusion of interaction terms.

**Fig. 1:**
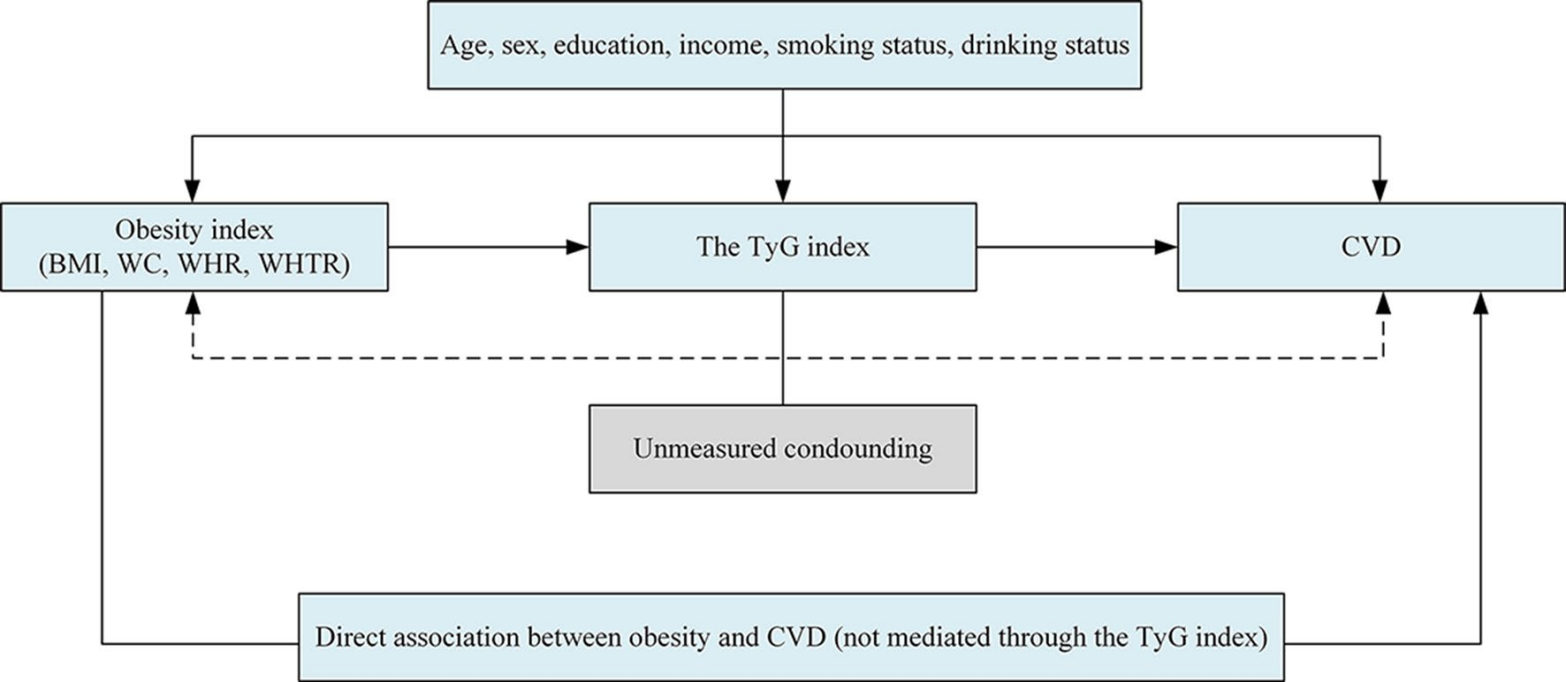
Causal diagram on the possible other mechanisms which the association of general and central obesity and CVD is mediated. All statistical models were based on this structure and were adjusted for age, sex, education, income, smoking status and drinking status. Because blood pressure, cholesterol and other metabolic factors presented alternative pathways potentially mediating parts of the total association, these variables were not entered as covariates in our models. The possibility of unmeasured confounding, which can never be ruled out in observational research, is indicated with dashed arrows. *BMI* body mass index, *CVD* cardiovascular disease, *TyG index* triglyceride-glucose index, *WC* Waist circumference, *WHR* waist-hip ratio, *WHTR* waist-height ratio

The assuming association between variables is illustrated in Fig. 1. [25] The VanderWeele’s method decomposes the total effect of general and central obesity on CVD (expressed as the hazard ratio [HR] vs the reference normal weight) into 2 components: the natural indirect effect size (ie, the effect size of general and central obesity mediated through the TyG index, and natural direct effect size (ie, the effect size of general and central obesity not explained through the TyG index) [26, 27]. Because these estimates are based on observational data, we term the estimates as total, indirect, and direct associations. The proportion of the association of general and central obesity and CVD mediated through the TyG index as a measure of the contribution of the natural indirect association with the total association was calculated on the log-transformed HR scale as log (indirect association HR)/log (total association HR), since HRs are assistive on this scale [28].

Sensitivity analyses were performed to test the robust of the associations. First, we excluded participants with baseline FBG ≥ 7 mmol/L. Second, the exposure and confounders were considered before the mediator at intervals of least two years (the exposure and confounders of baseline, the mediator of the second visit-up). Third, the confounders were considered before the mediator and exposure. Fourth, we included interaction between obesity parameters with the TyG index into the model. We have estimated excess risk mediated by insulin resistance measured by the TyG index by calculating percentage attenuation in the β coefficient of model with obesity parameters after inclusion mediator in the model as follows: Percentage of excess risk mediated (PERM) by the TyG index = 100* (confounder adjusted β_1_-confounder and the TyG index adjusted β_2_) / confounder adjusted β_1_, as previously reported. [29] Subgroup analysis stratified by age (< 60 vs ≥ 60 years) and sex was also performed. All analyses were conducted using SAS version 9.4 (SAS Institute Inc., Cary, NC, USA). A two-sided *P* < 0.05 was considered statistically significant.

## Results

### Baseline characteristics

The final sample included 94,136 participants. The mean age of the population was 51.24 ± 12.37 years. During a median follow-up of 13.01 years, we identified 7327 (7.78%) cases of incident CVD, including 5898 (6.27%) stroke and 1622 (1.72%) MI. baseline characteristics of the study population are presented in Table 1. There was a significant difference between the incident CVD and non-CVD group in age, sex, education, income, smoking, drinking, hypertension, diabetes, dyslipidemia, medications, SBP, DBP, FBG, TC, TG, HDL-C, LDL-C, and hs-CRP. The BMI, WC, WHR, WHTR, proportion of obesity and the TyG index was higher in participants with CVD than that without CVD.

**Table 1:** Baseline characteristics according to incident CVD status

Characteristics	Total (n = 94,136)	No incident CVD (n = 86,809)	Incident CVD (n = 7327)	*P* value
Age, years	51.24 ± 12.37	50.68 ± 12.39	57.79 ± 10.13	< 0.0001
Men, n (%)	74,776 (79.43)	68,246 (78.62)	6530 (89.12)	< 0.0001
High school or above, n (%)	18,597 (20.16)	17,762 (20.85)	835 (11.81)	< 0.0001
Income > 800 yuan/month, n (%)	13,107 (14.22)	12,226 (14.36)	881 (12.50)	< 0.0001
Current smoker, n (%)	31,700 (34.38)	28,992 (34.05)	2708 (38.33)	< 0.0001
Current alcohol use, n (%)	34,533 (37.44)	31,952 (37.51)	2581 (36.55)	0.1084
Hypertension, n (%)	10,844 (11.52)	9261 (10.67)	1583 (21.61)	< 0.0001
Diabetes Mellitus, n (%)	2684 (2.85)	2227 (2.56)	457 (6.24)	< 0.0001
Dyslipidemia, n (%)	5033 (5.35)	4457 (5.13)	576 (7.86)	< 0.0001
Antihypertensive drugs, n (%)	9347 (9.93)	7959 (9.17)	1388 (18.94)	< 0.0001
Antidiabetic drugs, n (%)	2044 (2.17)	1690 (1.95)	354 (4.83)	< 0.0001
Lipid-lowering drugs, n (%)	703 (0.75)	629 (0.72)	74 (1.01)	0.0064
Body mass index, kg/m^2^	25.02 ± 3.49	24.96 ± 3.49	25.71 ± 3.47	< 0.0001
Underweight	1714 (1.82)	1653 (1.90)	61 (0.83)	< 0.0001
Normal weight	47,177 (50.12)	44,051 (50.74)	3126 (42.66)	< 0.0001
Overweight	27,952 (29.69)	25,531 (29.41)	2421 (33.04)	
Obesity	17,293 (18.37)	15,574 (17.94)	1719 (23.46)	
Waist circumference, cm	86.89 ± 10.03	86.63 ± 10.03	89.94 ± 9.53	< 0.0001
Normal	54,710 (58.12)	51,296 (59.09)	3414 (46.59)	< 0.0001
Obesity	39,426 (41.88)	35,513 (40.91)	3913 (53.41)	
Waist-hip ratio	0.89 ± 0.07	0.89 ± 0.07	0.91 ± 0.07	< 0.0001
Normal	40,915 (43.46)	38,143 (43.94)	2772 (37.83)	< 0.0001
Obesity	53,221 (56.54)	48,666 (56.06)	4555 (62.17)	
Waist-height ratio	0.52 ± 0.06	0.52 ± 0.06	0.54 ± 0.06	< 0.0001
Normal	85,834 (91.18)	79,479 (91.56)	6355 (86.73)	< 0.0001
Obesity	8302 (8.82)	7330 (8.44)	972 (13.27)	
Systolic blood pressure, mm Hg	130.62 ± 20.89	129.63 ± 20.40	142.34 ± 22.87	< 0.0001
Diastolic blood pressure, mm Hg	83.45 ± 11.77	83.04 ± 11.58	88.33 ± 12.82	< 0.0001
Fasting plasma glucose, mmol/L	5.47 ± 1.67	5.43 ± 1.61	5.92 ± 2.20	< 0.0001
Total cholesterol, mmol/L	4.95 ± 1.15	4.93 ± 1.14	5.11 ± 1.19	< 0.0001
Triglycerides, mmol/L	1.67 ± 1.38	1.66 ± 1.37	1.88 ± 1.50	< 0.0001
High sensitivity C-reactive protein, mg/dL	2.38 ± 6.47	2.33 ± 6.48	3.05 ± 6.35	< 0.0001
Triglyceride-glucose index	8.65 ± 0.70	8.64 ± 0.69	8.83 ± 0.72	< 0.0001

*CVD* cardiovascular disease

### Correlation between the obesity indexes with the TyG index

Both the general central obesity indexes were significant with the TyG index (Additional file 1: Figure S2). R^2^ from the linear regression for the correlation between BMI and the TyG index was 0.1088, indicating 10.88% of the variation in the BMI was explained by variation in the TyG index in this model. Similar results were observed for WC, WHR, and WHTR.

### Mediation analysis

**Fig. 2:**
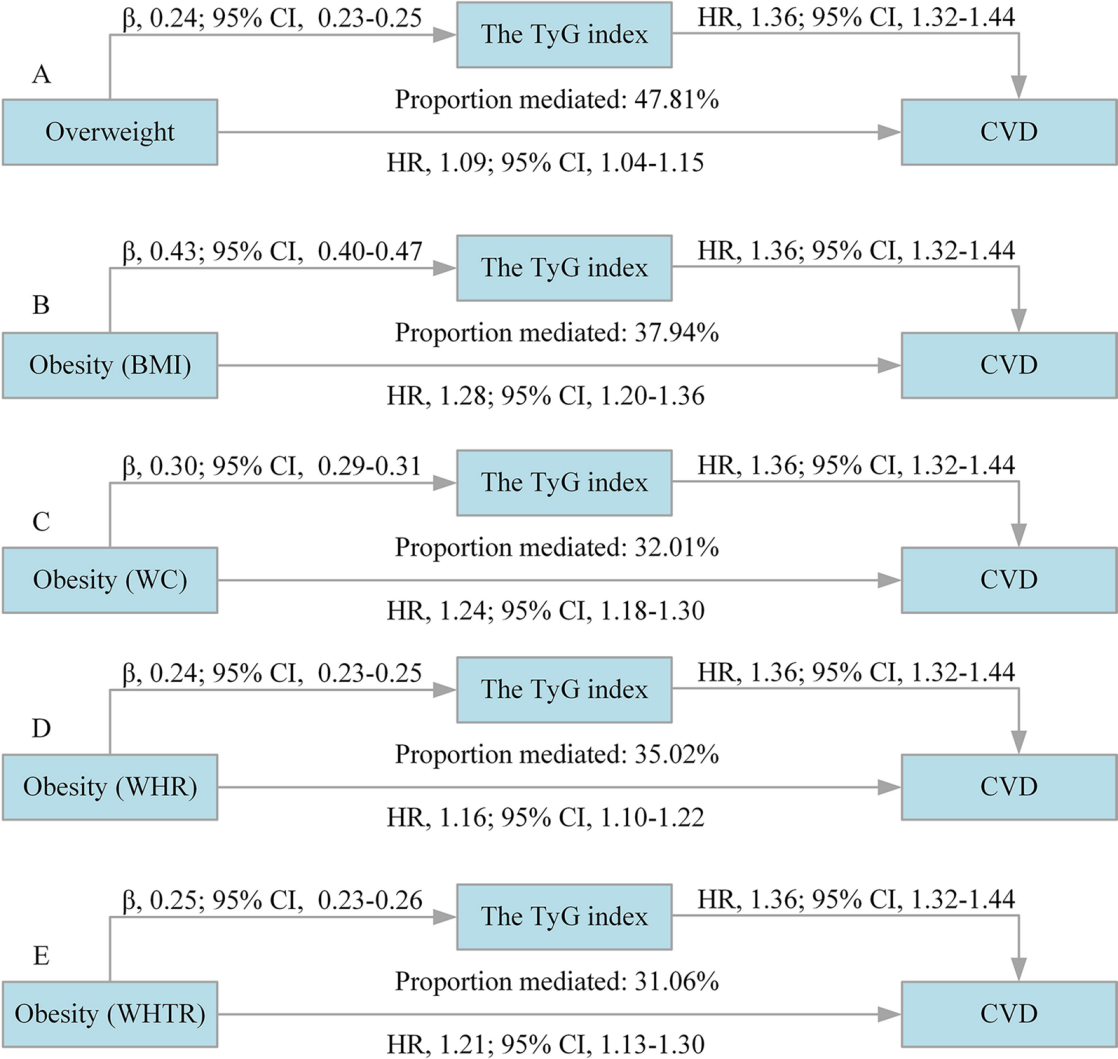
Decomposition of the total association of general and central obesity and the risk of CVD into direct and indirect associations mediated by the TyG index. *BMI* body mass index, *CI* confidence interval, *CVD* cardiovascular disease, *HR* hazard ratio, *TyG* triglyceride-glucose index, *WC* waist circumference, *WHR* waist circumference to hip ratio, *WHTR* waist circumference to height ratio. Compared with normal weight participants for general obesity and WC < 90 cm in men or < 85 cm in women, WHR < 0.90 in men or < 0.80 in women, and WHTR < 0.60 as a reference for central obesity. All models were adjusted for age, sex, education, income, smoking status, drinking status, history of hypertension, diabetes, dyslipidemia, antihypertensive agents, antidiabetic agents, lipide-lowering agents, systolic blood pressure, diastolic blood pressure, total cholesterol, high density lipoprotein cholesterol, and high sensitivity C-reactive protein

Results on the decomposition of the total association of general and central obesity with the risk of CVD into direct and indirect associations mediated by the TyG index is shown as Table 2 and Fig. 2. Mediation analysis showed that the risk of CVD increased 18% (HR [total association], 1.18; 95% confidence interval [CI], 1.12–1.24) for overweight vs the reference normal weight, which increased to 45% (HR, 1.45; 95% CI, 1.36–1.54) for the obesity group, of which 47.81% and 37.94% was mediated through the TyG index, the HR for the indirect association was 1.07 (95% CI, 1.07–1.09) and 1.13 (95% CI, 1.11–1.15) for overweight and obesity respectively.

**Table 2:** Decomposition of the total association of general and central obesity and the risk of CVD into direct and indirect associations mediated by the TyG index

Exposures	Association^a^						Proportion mediated, %
	Total effect^b^		Indirect effect		Direct effect		
	HR (95% CI)	*P* value	HR (95% CI)	*P* value	HR (95% CI)	*P* value	
General obesity							
Overweight (25 ≤ BMI < 28 kg/m^2^)	1.18 (1.12–1.24)	< 0.0001	1.07 (1.07–1.09)	< 0.0001	1.09 (1.04–1.15)	< 0.0001	47.81
Obesity (BMI ≥ 28 kg/m^2^)	1.45 (1.36–1.54)	< 0.0001	1.13 (1.11–1.15)	< 0.0001	1.28 (1.20–1.36)	< 0.0001	37.94
Central obesity							
WC ≥ 90 cm in men or ≥ 85 cm in women	1.35 (1.29–1.41)	< 0.0001	1.09 (1.08–1.10)	< 0.0001	1.24 (1.18–1.30)	< 0.0001	32.01
WHR ≥ 0.90 in men or ≥ 0.80 in women	1.24 (1.19–1.30)	< 0.0001	1.07 (1.06–1.08)	< 0.0001	1.16 (1.10–1.22)	< 0.0001	35.02
WHTR ≥ 0.60	1.30 (1.22–1.40)	< 0.0001	1.08 (1.07–1.09)	< 0.0001	1.21 (1.13–1.30)	< 0.0001	31.06

All models were adjusted for age, sex, education, income, smoking status, drinking status, history of hypertension, diabetes, dyslipidemia, antihypertensive agents, antidiabetic agents, lipide-lowering agents, systolic blood pressure, diastolic blood pressure, total cholesterol, high density lipoprotein cholesterol, and high sensitivity C-reactive protein

*BMI* body mass index, *CI* confidence interval, *CVD* cardiovascular disease, *HR* hazard ratio, *TyG* triglyceride-glucose index, *WC* waist circumference, *WHR* waist circumference to hip ratio, *WHTR* waist circumference to height ratio

^a^Compared with normal weight participants for general obesity and WC < 90 cm in men or < 85 cm in women, WHR < 0.90 in men or < 0.80 in women, and WHTR < 0.60 as a reference for central obesity

^b^Decomposition of total associations into natural indirect and natural direct associations was done according to the 2-stage regression method proposed by VanderWeele and performed with the SAS macro provided by ValerWeele. Confidence intervals were calculated according to the delta method procedure

On scrutinizing the relationship between central obesity and CVD, the results yielded a total association HR of 1.35 (1.29–1.41) for WC ≥ 90 cm in men or ≥ 85 cm in women, 1.24 (95% CI, 1.19–1.30) for WHR ≥ 0.90 in men or ≥ 0.80 in women, and 1.30 (95% CI, 1.22–1.40) for WHTR ≥ 0.60, compared with their counterparts with normal weight, and the proportion mediated was 32.01%, 35.02%, and 31.06% respectively (Table 2 and Fig. 2). In the subtype analyses of CVD, similar results were yield for stroke and MI (Additional file 1: Table S1 and S2).

### Sensitivity and subgroup analysis

The results of the sensitivity analysis performed in this study showed that excluding participants with baseline FBG ≥ 7 mmol/L (n = 7642) slightly attenuated the indirect association of general and central obesity with CVD, the proportion mediated by the TyG index was 36.35% for overweight, 26.96% for general obesity, and 23.69%, 29.45%, 21.57% for WC, WHR and WHTR as the index of central obesity. Sensitivity analyses by adding a lag time (a two-year lag time in this study) between the general and central obesity and the TyG index, applying a two-year period between the confounders and general and central obesity, in addition to adding interaction terms into the models presented the same pattern of results, with a decreased proportion mediated compared with the main results (Table 3, Additional file 1: Table S3 and S4). A substantial attenuation of HRs for BMI (44.84% for overweight and 32.54% for obesity), WC (28.04%), WHR (30.44%), and WHTR (27.49%) and incident CVD was observed after adjustment the TyG index, suggesting that a significant part of the excess risk was attributable to the TyG index (Additional file 1: Table S5). Subgroup analyses revealed similar results across the young and elderly participants (Additional file 1: Table S6), men and women (Additional file 1: Table S7).

**Table 3:** Sensitivity analysis on the decomposition of the total association of general and central obesity and the risk of CVD into direct and indirect associations mediated by the TyG index

Exposures	Association^a^						Proportion mediated, %
	Total effect^b^		Indirect effect		Direct effect		
	HR (95% CI)	*P* value	HR (95% CI)	*P* value	HR (95% CI)	*P* value	
Sensitivity analysis 1: Excluding participants with baseline FBG ≥ 7 mmol/L							
General obesity							
Overweight (25 ≤ BMI < 28, kg/m^2^)	1.18 (1.12–1.26)	< 0.0001	1.06 (1.05–1.07)	< 0.0001	1.12 (1.06–1.18)	< 0.0001	36.35
Obesity (BMI ≥ 28 kg/m^2^)	1.49 (1.40–1.59)	< 0.0001	1.10 (1.08–1.12)	< 0.0001	1.36 (1.27–1.46)	< 0.0001	26.96
Central obesity							
WC ≥ 90 cm in men or ≥ 85 cm in women	1.35 (1.29–1.43)	< 0.0001	1.07 (1.06–1.08)	< 0.0001	1.27 (1.21–1.34)	< 0.0001	23.69
WHR ≥ 0.90 in men or ≥ 0.80 in women	1.22 (1.16–1.29)	< 0.0001	1.06 (1.05–1.07)	< 0.0001	1.16 (1.10–1.22)	< 0.0001	29.45
WHTR ≥ 0.60	1.33 (1.23–1.44)	< 0.0001	1.06 (1.05–1.07)	< 0.0001	1.26 (1.17–1.36)	< 0.0001	21.57
Sensitivity analysis 2: Adding a lag time between exposures and mediator							
General obesity							
Overweight (25 ≤ BMI < 28, kg/m^2^)	1.22 (1.15–1.30)	< 0.0001	1.07 (1.06–1.08)	< 0.0001	1.14 (1.07–1.22)	< 0.0001	36.15
Obesity (BMI ≥ 28 kg/m^2^)	1.49 (1.39–1.60)	< 0.0001	1.13 (1.10–1.15)	< 0.0001	1.33 (1.23–1.43)	< 0.0001	34.02
Central obesity							
WC ≥ 90 cm in men or ≥ 85 cm in women	1.33 (1.26–1.40)	< 0.0001	1.07 (1.06–1.08)	< 0.0001	1.24 (1.17–1.31)	< 0.0001	27.85
WHR ≥ 0.90 in men or ≥ 0.80 in women	1.23 (1.16–1.30)	< 0.0001	1.06 (1.05–1.07)	< 0.0001	1.15 (1.09–1.22)	< 0.0001	32.32
WHTR ≥ 0.60	1.30 (1.20–1.41)	< 0.0001	1.06 (1.05–1.07)	< 0.0001	1.22 (1.13–1.33)	< 0.0001	25.39
Sensitivity analysis 3: Adding a lag time between the confounders and set of exposures and mediator							
General obesity							
Overweight (25 ≤ BMI < 28, kg/m^2^)	1.16 (1.09–1.23)	< 0.0001	1.07 (1.06–1.08)	< 0.0001	1.08 (1.02–1.15)	0.0102	46.37
Obesity (BMI ≥ 28 kg/m^2^)	1.40 (1.30–1.51)	< 0.0001	1.13 (1.11–1.16)	< 0.0001	1.24 (1.14–1.33)	< 0.0001	41.32
Central obesity							
WC ≥ 90 cm in men or ≥ 85 cm in women	1.32 (1.25–1.39)	< 0.0001	1.07 (1.06–1.08)	< 0.0001	1.23 (1.17–1.30)	< 0.0001	26.63
WHR ≥ 0.90 in men or ≥ 0.80 in women	1.13 (1.07–1.19)	< 0.0001	1.04 (1.03–1.04)	< 0.0001	1.09 (1.03–1.15)	0.0038	33.04
WHTR ≥ 0.60	1.42 (1.30–1.55)	< 0.0001	1.08 (1.07–1.09)	< 0.0001	1.31 (1.20–1.43)	< 0.0001	25.13
Sensitivity analysis 4: Adding interaction terms into the models							
General obesity							
Overweight (25 ≤ BMI < 28, kg/m^2^)	1.18 (1.12–1.25)	< 0.0001	1.07 (1.06–1.09)	< 0.0001	1.10 (1.04–1.17)	< 0.0001	34.68
Obesity (BMI ≥ 28 kg/m^2^)	1.45 (1.16–1.82)	0.0012	1.09 (1.06–1.12)	< 0.0001	1.33 (1.06–1.68)	0.0142	30.52
Central obesity							
WC ≥ 90 cm in men or ≥ 85 cm in women	1.35 (1.14–1.60)	0.0004	1.08 (1.06–1.09)	< 0.0001	1.25 (1.06–1.48)	0.0080	39.07
WHR ≥ 0.90 in men or ≥ 0.80 in women	1.25 (1.14–1.36)	< 0.0001	1.07 (1.06–1.08)	< 0.0001	1.16 (1.06–1.28)	0.0010	30.00
WHTR ≥ 0.60	1.31 (1.22–1.40)	< 0.0001	1.08 (1.05–1.10)	< 0.0001	1.21 (1.13–1.31)	< 0.0001	27.43

All models were adjusted for age, sex, education, income, smoking status, drinking status, history of hypertension, diabetes, dyslipidemia, antihypertensive agents, antidiabetic agents, lipide-lowering agents, systolic blood pressure, diastolic blood pressure, total cholesterol, high density lipoprotein cholesterol, and high sensitivity C-reactive protein

*BMI* body mass index, *CI* confidence interval, *CVD* cardiovascular disease, *HR* hazard ratio, *TyG* triglyceride-glucose index, *WC* waist circumference, *WHR* waist circumference to hip ratio, *WHTR* waist circumference to height ratio

^a^Compared with normal weight participants for general obesity and WC < 90 cm in men or < 85 cm in women, WHR < 0.90 in men or < 0.80 in women, and WHTR < 0.60 as a reference for central obesity

^b^Decomposition of total associations into natural indirect and natural direct associations was done according to the 2-stage regression method proposed by VanderWeele and performed with the SAS macro provided by ValerWeele. Confidence intervals were calculated according to the delta method procedure

## Discussion

In this large prospective population-based cohort study, we found that the association of general and central obesity with CVD was mediated through the TyG index. Similar patterns were observed for stroke and MI. The trend remained robust among stratified analyses and multiple sensitivity analyses. These findings suggested public health efforts aiming at the reduction of body weight might decrease the sequelae of insulin resistance and the burden of CVD.

Obesity is frequently associated with elevated risk of CVD. Both observational [30] and Mendelian randomization approaches [31] have consistently shown the association between general obesity and CVD risk. In addition to the important clinical implications of BMI assessment, studies also proved that central obesity (measured by WC, WHR, and WHTR) is also an important risk factor for various obesity-related chronic diseases [32]. Consistent with these study, our study also demonstrated a significant relationship of general and central obesity with the risk of CVD, even after adjustment for the TyG index. Obesity can increase CVD morbidity and mortality directly and indirectly. Direct effects are mediated by obesity-induced structural and functional adaptions of the cardiovascular system to accommodate excess body weight, as well as by adipocyte effects on inflammation and vascular homeostasis, leading to a pro-inflammatory and pro-thrombotic milieu. Indirect effects are mediated by concomitant CVD risk factors. Previous study showed that obesity is a well-established risk factor of insulin resistance, individuals who are overweight or obese are more likely to develop insulin resistance indicating early impaired glucose metabolism [33]. Epidemiological studies have shown a significant association of insulin resistance with CVD independent of diabetes, which was greater in the presence of general obesity [34]. Therefore, insulin resistance might be a potential important mediator of the association between obesity and the risk of CVD.

Our study results provide epidemiologic support for the biologically plausible hypothesis that insulin resistance plays an important role in the pathway between obesity and CVD, the proportion mediated of the TyG index was 47.81% for t overweight, 37.94% for general obesity, 32.01%, 35.02%, and 31.06% for central obesity measured by WC, WHR, and WHTR, respectively. Insulin resistance often clusters with various classical risk factors such as lipid abnormalities, glucose intolerance, and high blood pressure, which have been confirmed to mediate the effect of general and central obesity on CVD in several previous studies [35, 36]. While the role of insulin resistance in the relationship between obesity and CVD has not been established well previously. A retrospective cohort analysis of 6078 participants aged over 60 years revealed the TyG index mediated the effect of BMI on CVD event, HR for the indirect effect through the TyG index was 1.01, but the proportion mediated was not provided in this study, and the obesity index was only restricted to BMI [37]. In contrast, our prospective analysis with a large sample size (N = 94,136) can provided a stronger statistical power, focused on a series of indices on both general and central obesity, and used a newly developed method to quantify the proportion mediated of the TyG index. This approach offers several advantages over conventional methods. In particular, it can accommodate interactions between BMI and its mediators by incorporating interaction terms into the outcome model and using a separate regression model for each exposure–mediator association [38]. In addition, conventional methods often overlook mediator-outcome confounders and this can introduce bias in the direct and indirect effect estimates [36]. We adjusted for confounders for both obesity-CVD and mediator-CVD in regression that parameterized those association. Our results together with the previous findings indicated that controlling the TyG index may can be effective to reduce the effects of general and central obesity on CVD.

Our result also revealed that the TyG index explained more to the association between general obesity and CVD than central obesity, which was indirectly supported by the evidence that central obesity plays a more importance role than BMI in determining the risk of CVD [32]. In the sensitivity analysis, we found after excluded those with FBG ≥ 7 mmol/L, the proportion mediated was attenuated slightly, indicating general and central obesity contributed more to the risk of CVD among individuals with normal FBG levels. This is in line with the results from the National Sample Cohort (NSC) database, which showed a higher BMI on the risk of CVD event was significantly weaker among individuals with diabetes than those with without diabetes [39]. It is conceivable that the association between obesity and CVD mediated through the TyG index can in part be explained by the development of diabetes. For public health programs, the mediating role of the TyG index in the association of general and central obesity and CVD indicates that it is important to emphasize body weight management and to encourage individuals to focus on their body fat distribution as well as the level of FBG.

Overweight and obesity (general and central obesity) have harmful effects on CVD risk, and the underlying mechanism has been thoroughly investigated [40]. Adipose tissue increases basal lipolysis and releases free fatty acids (FFA), interleukins and cytokines that drive cardiac dysfunction by accelerating atherosclerotic processes and modifying factors associated with inflammation and endothelial and coagulation dysfunction [41]. The increase in FFA due to obesity can trigger insulin resistance, which further inhibits insulin signaling and insulin-stimulated glucose uptake in skeletal muscles and increases glucose delivery by the liver [42]. Additionally, positive energy balance results in adipocyte hypertrophy and ectopic accumulation that leads to metabolic abnormalities, such as insulin resistance and beta-cell dysfunction [43]. Furthermore, inflammatory factors associated with obesity promote the processes of lipolysis and hepatic triglyceride synthesis, as well as hyperlipidemia induced by increased fatty acid esterification [9]. Insulin resistance is itself also associated with obesity, both the obesity and insulin resistance could enhance the risk of CVD. Hence, the TyG index is a plausible link between obesity and the risk of CVD.

Our findings have clinical and public health implications. In fact, behavioral interventions which are intended to properly manage and control weight gain are successful only for a short time [44]. Since the majority of weight-loss drugs have no effectiveness and efficiency, and numerous surgical procedures are performed for obese individuals, the global occurrence of CVDs has been regarded as a most important concern and challenge [45]. Efficient clinical and public health interventions aiming at imperative reductions in the predominance of some risk factors is necessary, since the prevalence of obesity is still growing during the past decades. The findings of our study supported the indirectly causal pathway way from obesity to insulin resistance and CVD, declaring that the proper control of insulin resistance can be effective to significantly reduce the effects of general and central obesity on CVD.

## Strengths and limitations

The strengths of the study include its prospective design, large community-based sample, and long follow-up period. Furthermore, mediation analysis is performed using a newly proposed 2-stage regression method for survival data, which is a mathematically consistent decomposition of the total association into direct and indirect associations with clear interpretations. However, the study also has several limitations. First, we only collected information on stroke and MI, and we may have underestimated the prevalence and incidence rates of CVD, which has broader subtypes (e.g., heart failure, coronary artery disease). Second, this is not a national representative sample; the results need to be interpreted with caution, particularly regarding generalizability. The prevalence of general and central obesity was higher in our study population than in the general Chinese population. Findings of this study may not be generalizable to other populations, as results were based on just the Kailuan community, in which approximately one-third of the participants were coal miners, who had different lifestyles and working environments than the rest of the participant. Third, although the possibility of unmeasured confounding cannot be ruled out, the magnitude of the observed effect sizes makes it unlikely that unmeasured confounding could completely explain our observed associations. Finally, data on insulin resistance and glycosylated hemoglobin (HbA1c) were not collected due to the large sample size and the high cost, we could not use homeostasis model assessment of insulin resistance (HOMA-IR) or HbA1c to reflect insulin resistance, which warrant further investigations.

## Conclusions

Our findings suggested that the association of general and central obesity with risk of CVD was mediated through the TyG index. Proper control of insulin resistance can be effective to significantly reduce the effects of general and central obesity on CVD.

## Supplementary Information

**Additional file 1: Table S1.** Decomposition of the total association between obesity indexes and the risk of stroke into direct and indirect associations mediated by the TyG index. **Table S2.** Decomposition of the total association between obesity indexes and the risk of myocardial infarction into direct and indirect associations mediated by the TyG index. **Table S3.** Sensitivity analysis on the decomposition of the total association between obesity indexes and the risk of stroke into direct and indirect associations mediated by the TyG index. **Table S4.** Sensitivity analysis on the decomposition of the total association between obesity indexes and the risk of myocardial infarction into direct and indirect associations mediated by the TyG index. **Table S5.** Percentage excess risk mediated by the TyG index. **Table S6.** Decomposition of the total association between obesity indexes and the risk of CVD into direct and indirect associations mediated by the TyG index stratified by age. **Table S7.** Decomposition of the total association between obesity indexes and the risk of CVD into direct and indirect associations mediated by the TyG index stratified by sex. **Figure S1.** Flowchart of the study. **Figure S2.** Correlation between obesity index and the TyG index.

## Data Availability

The datasets used and/or analyzed during the current study are available from the corresponding author on reasonable request.

## References

[1] Iliodromiti S, Celis-Morales CA, Lyall DM, Anderson J, Gray SR, Mackay DF, Nelson SM, Welsh P, Pell JP, Gill JMR (2018). The impact of confounding on the associations of different adiposity measures with the incidence of cardiovascular disease: a cohort study of 296 535 adults of white European descent. Eur Heart J.

[2] Kelly T, Yang W, Chen CS, Reynolds K, He J (2008). Global burden of obesity in 2005 and projections to 2030. Int J Obes (Lond).

[3] Wormser D, Kaptoge S, Di Angelantonio E, Wood AM, Pennells L, Thompson A, Sarwar N, Kizer JR, Lawlor DA, Nordestgaard BG (2011). Separate and combined associations of body-mass index and abdominal adiposity with cardiovascular disease: collaborative analysis of 58 prospective studies. Lancet.

[4] Yusuf S, Anand S (2011). Body-mass index, abdominal adiposity, and cardiovascular risk. Lancet.

[5] Khan SS, Ning H, Wilkins JT, Allen N, Carnethon M, Berry JD, Sweis RN, Lloyd-Jones DM (2018). Association of body mass index with lifetime risk of cardiovascular disease and compression of morbidity. JAMA Cardiol.

[6] Global, regional, and national age-sex-specific mortality for 282 causes of death in 195 countries and territories, 1980–2017: a systematic analysis for the Global Burden of Disease Study 2017. Lancet (London, England) 2018, 392 (10159):1736–1788. 10.1016/S0140-6736(18)32203-7 PMC6227606 30496103

[7] Steppan CM, Bailey ST, Bhat S, Brown EJ, Banerjee RR, Wright CM, Patel HR, Ahima RS, Lazar MA (2001). The hormone resistin links obesity to diabetes. Nature.

[8] Min J, Weitian Z, Peng C, Yan P, Bo Z, Yan W, Yun B, Xukai W (2016). Correlation between insulin-induced estrogen receptor methylation and atherosclerosis. Cardiovasc Diabetol.

[9] Ormazabal V, Nair S, Elfeky O, Aguayo C, Salomon C, Zuñiga FA (2018). Association between insulin resistance and the development of cardiovascular disease. Cardiovasc Diabetol.

[10] Guerrero-Romero F, Simental-Mendía LE, González-Ortiz M, Martínez-Abundis E, Ramos-Zavala MG, Hernández-González SO, Jacques-Camarena O, Rodríguez-Morán M (2010). The product of triglycerides and glucose, a simple measure of insulin sensitivity Comparison with the euglycemic-hyperinsulinemic clamp. J Clin Endocrinol Metab..

[11] Du T, Yuan G, Zhang M, Zhou X, Sun X, Yu X (2014). Clinical usefulness of lipid ratios, visceral adiposity indicators, and the triglycerides and glucose index as risk markers of insulin resistance. Cardiovasc Diabetol.

[12] Zhang M, Wang B, Liu Y, Sun X, Luo X, Wang C, Li L, Zhang L, Ren Y, Zhao Y (2017). Cumulative increased risk of incident type 2 diabetes mellitus with increasing triglyceride glucose index in normal-weight people: The Rural Chinese Cohort Study. Cardiovasc Diabetol.

[13] Lee SB, Ahn CW, Lee BK, Kang S, Nam JS, You JH, Kim MJ, Kim MK, Park JS (2018). Association between triglyceride glucose index and arterial stiffness in Korean adults. Cardiovasc Diabetol.

[14] Barzegar N, Tohidi M, Hasheminia M, Azizi F, Hadaegh F (2020). The impact of triglyceride-glucose index on incident cardiovascular events during 16 years of follow-up: Tehran Lipid and Glucose Study. Cardiovasc Diabetol.

[15] Jin C, Chen S, Vaidya A, Wu Y, Wu Z, Hu FB, Kris-Etherton P, Wu S, Gao X (2017). Longitudinal change in fasting blood glucose and myocardial infarction risk in a population without diabetes. Diabetes Care.

[16] Wang C, Yuan Y, Zheng M, Pan A, Wang M, Zhao M, Li Y, Yao S, Chen S, Wu S (2020). Association of age of onset of hypertension with cardiovascular diseases and mortality. J Am Coll Cardiol.

[17] Tian X, Zuo Y, Chen S, Li H, He Y, Zhang L, An J, Wu S, Luo Y, Wang A (2020). Association of changes in lipids with risk of myocardial infarction among people without lipid-lowering therapy. Atherosclerosis.

[18] Yang S, Liu M, Wang S, Jia W, Han K, He Y (2020). Waist-calf circumference ratio is an independent risk factor of HRQoL in centenarians. Diabetes Metab Syndr Obes.

[19] Tian X, Zuo Y, Chen S, Liu Q, Tao B, Wu S, Wang A (2021). Triglyceride-glucose index is associated with the risk of myocardial infarction: an 11-year prospective study in the Kailuan cohort. Cardiovasc Diabetol.

[20] Wang A, Wang G, Liu Q, Zuo Y, Chen S, Tao B, Tian X, Wang P, Meng X, Wu S (2021). Triglyceride-glucose index and the risk of stroke and its subtypes in the general population: an 11-year follow-up. Cardiovasc Diabetol.

[21] Wu S, An S, Li W, Lichtenstein AH, Gao J, Kris-Etherton PM, Wu Y, Jin C, Huang S, Hu FB (2019). Association of trajectory of cardiovascular health score and incident cardiovascular disease. JAMA Netw Open.

[22] Stroke--1989. Recommendations on stroke prevention, diagnosis, and therapy. Report of the WHO Task Force on Stroke and other Cerebrovascular Disorders. *Stroke.* 1989, 20 (10):1407–1431. 10.1161/01.str.20.10.1407 2799873

[23] Thygesen K, Alpert JS, Jaffe AS, Chaitman BR, Bax JJ, Morrow DA, White HD (2018). Fourth universal definition of myocardial infarction (2018). J Am Coll Cardiol.

[24] VanderWeele TJ (2011). Causal mediation analysis with survival data. Epidemiology.

[25] Greenland S, Pearl J, Robins JM (1999). Causal diagrams for epidemiologic research. Epidemiology.

[26] Lange T, Hansen KW, Sørensen R, Galatius S (2017). Applied mediation analyses: a review and tutorial. Epidemiol Health.

[27] VanderWeele TJ (2016). Mediation analysis: a practitioner's guide. Annu Rev Public Health.

[28] Valeri L, Vanderweele TJ (2013). Mediation analysis allowing for exposure-mediator interactions and causal interpretation: theoretical assumptions and implementation with SAS and SPSS macros. Psychol Methods.

[29] Tabák AG, Brunner EJ, Lindbohm JV, Singh-Manoux A, Shipley MJ, Sattar N, Kivimäki M (2022). Risk of macrovascular and microvascular disease in diabetes diagnosed using oral glucose tolerance test with and without confirmation by hemoglobin A1c: the Whitehall II cohort study. Circulation.

[30] Canoy D, Cairns BJ, Balkwill A, Wright FL, Green J, Reeves G, Beral V (2013). Body mass index and incident coronary heart disease in women: a population-based prospective study. BMC Med.

[31] Nordestgaard BG, Palmer TM, Benn M, Zacho J, Tybjaerg-Hansen A, Davey Smith G, Timpson NJ (2012). The effect of elevated body mass index on ischemic heart disease risk: causal estimates from a Mendelian randomisation approach. PLoS Med.

[32] Wang Z, Hoy WE (2004). Waist circumference, body mass index, hip circumference and waist-to-hip ratio as predictors of cardiovascular disease in Aboriginal people. Eur J Clin Nutr.

[33] Liao C, Gao W, Cao W, Lv J, Yu C, Wang S, Pang Z, Cong L, Wang H, Wu X (2021). Associations of metabolic/obesity phenotypes with insulin resistance and C-reactive protein: results from the CNTR study. Diabetes Metab Syndr Obes.

[34] Zhao Q, Zhang TY, Cheng YJ, Ma Y, Xu YK, Yang JQ, Zhou YJ (2020). Impacts of triglyceride-glucose index on prognosis of patients with type 2 diabetes mellitus and non-ST-segment elevation acute coronary syndrome: results from an observational cohort study in China. Cardiovasc Diabetol.

[35] Bakhtiyari M, Schmidt N, Hadaegh F, Khalili D, Mansournia N, Asgari S, Mansournia MA (2018). Direct and indirect effects of central and general adiposity on cardiovascular diseases: the Tehran Lipid and Glucose Study. Eur J Prev Cardiol.

[36] Lu Y, Hajifathalian K, Rimm EB, Ezzati M, Danaei G (2015). Mediators of the effect of body mass index on coronary heart disease: decomposing direct and indirect effects. Epidemiology.

[37] Li S, Guo B, Chen H, Shi Z, Li Y, Tian Q, Shi S (2019). The role of the triglyceride (triacylglycerol) glucose index in the development of cardiovascular events: a retrospective cohort analysis. Sci Rep.

[38] Kaufman JS, Maclehose RF, Kaufman S (2004). A further critique of the analytic strategy of adjusting for covariates to identify biologic mediation. Epidemiol Perspect Innov.

[39] Kong KA, Park J, Hong SH, Hong YS, Sung YA, Lee H (2017). Associations between body mass index and mortality or cardiovascular events in a general Korean population. PLoS ONE.

[40] Van Gaal LF, Mertens IL, De Block CE (2006). Mechanisms linking obesity with cardiovascular disease. Nature.

[41] Grundy SM (2004). Obesity, metabolic syndrome, and cardiovascular disease. J Clin Endocrinol Metab.

[42] Sinha R, Dufour S, Petersen KF, LeBon V, Enoksson S, Ma YZ, Savoye M, Rothman DL, Shulman GI, Caprio S (2002). Assessment of skeletal muscle triglyceride content by (1)H nuclear magnetic resonance spectroscopy in lean and obese adolescents: relationships to insulin sensitivity, total body fat, and central adiposity. Diabetes.

[43] Lopez-Jimenez F, Almahmeed W, Bays H, Cuevas A, Di Angelantonio E, le Roux CW, Sattar N, Sun MC, Wittert G, Pinto FJ (2022). Obesity and cardiovascular disease: mechanistic insights and management strategies. A joint position paper by the World Heart Federation and World Obesity Federation. Eur J Prev Cardiol..

[44] Franz MJ, VanWormer JJ, Crain AL, Boucher JL, Histon T, Caplan W, Bowman JD, Pronk NP (2007). Weight-loss outcomes: a systematic review and meta-analysis of weight-loss clinical trials with a minimum 1-year follow-up. J Am Diet Assoc.

[45] Anand SS, Yusuf S (2011). Stemming the global tsunami of cardiovascular disease. Lancet.

