# Supplementary material for: Insulin resistance mediates obesity-related risk of cardiovascular disease: a prospective cohort study
Source: Cardiovasc Diabetol. 2022 Dec 23;21:289. doi: 10.1186/s12933-022-01729-9 (PMC9789633; doi:10.1186/s12933-022-01729-9)
Supplement: Supplementary file 1 — Additional file 1: Table S1. Decomposition of the total association between obesity indexes and the risk of stroke into direct and indirect associations mediated by the TyG index. Table S2. Decomposition of the total association between obesity indexes and the risk of myocardial infarction into direct and indirect associations mediated by the TyG index. Table S3. Sensitivity analysis on the decomposition of the total association between obesity indexes and the risk of stroke into direct and indirect associations mediated by the TyG index. Table S4. Sensitivity analysis on the decomposition of the total association between obesity indexes and the risk of myocardial infarction into direct and indirect associations mediated by the TyG index. Table S5. Percentage excess risk mediated by the TyG index. Table S6. Decomposition of the total association between obesity indexes and the risk of CVD into direct and indirect associations mediated by the TyG index stratified by age. Table S7. Decomposition of the total association between obesity indexes and the risk of CVD into direct and indirect associations mediated by the TyG index stratified by sex. Figure S1. Flowchart of the study. Figure S2. Correlation between obesity index and the TyG index. [file 12933_2022_1729_MOESM1_ESM.docx]

Table S1. Decomposition of the total association between obesity indexes and the risk of stroke into direct and indirect associations mediated by the TyG index

| Exposures | Association^a^ | | | | | | | | Proportion  mediated, % |
| --- | --- | --- | --- | --- | --- | --- | --- | --- | --- |
|  | Total effect^b^ |  |  | Indirect effect |  |  | Direct effect |  |  |
|  | HR(95% CI) | *P* value |  | HR(95% CI) | *P* value |  | HR(95% CI) | *P* value |  |
| General obesity |  |  |  |  |  |  |  |  |  |
| Overweight (25≤BMI<28, kg/m^2^) | 1.19(1.12-1.26) | <0.0001 |  | 1.07(1.06-1.08) | <0.0001 |  | 1.11(1.04-1.17) | 0.0007 | 42.08 |
| Obesity (BMI≥28kg/m^2^) | 1.41(1.32-1.51) | <0.0001 |  | 1.12(1.10-1.14) | <0.0001 |  | 1.26(1.18-1.35) | <0.0001 | 36.63 |
| Central obesity |  |  |  |  |  |  |  |  |  |
| WC≥90cm in men or ≥85cm in women | 1.31(1.24-1.38) | <0.0001 |  | 1.08(1.07-1.09) | <0.0001 |  | 1.21(1.15-1.27) | <0.0001 | 32.35 |
| WHR≥0.90 in men or ≥0.80 in women | 1.31(1.22-1.41) | <0.0001 |  | 1.07(1.06-1.08) | <0.0001 |  | 1.22(1.14-1.32) | <0.0001 | 27.92 |
| WHTR≥0.60 | 1.39(1.29-1.50) | <0.0001 |  | 1.09(1.08-1.10) | <0.0001 |  | 1.27(1.18-1.37) | <0.0001 | 30.22 |

Abbreviations, BMI, body mass index; CI, confidence interval; HR, hazard ratio; TyG, triglyceride-glucose index; WC, waist circumference; WHR, waist circumference to hip ratio; WHTR, waist circumference to height ratio.

^a^ Compared with normal weight participants for general obesity and WC<90cm in men and <85cm in women, or WHR<0.90 in men and <0.80 in women, or WHTR<0.60 as a reference for central obesity.

^b^ Decomposition of total associations into natural indirect and natural direct associations was done according to the 2-stage regression method proposed by VanderWeele and performed with the SAS macro provided by ValerWeele. Confidence intervals were calculated according to the delta method procedure.

All models were adjusted for age, sex, education, income, smoking status, drinking status, history of hypertension, diabetes, dyslipidemia, antihypertensive agents, antidiabetic agents, lipide-lowering agents, systolic blood pressure, diastolic blood pressure, total cholesterol, high density lipoprotein cholesterol, and high sensitivity C-reactive protein.

Table S2. Decomposition of the total association between obesity indexes and the risk of myocardial infarction into direct and indirect associations mediated by the TyG index

| Exposures | Association^a^ | | | | | | | | Proportion  mediated, % |
| --- | --- | --- | --- | --- | --- | --- | --- | --- | --- |
|  | Total effect^b^ |  |  | Indirect effect |  |  | Direct effect |  |  |
|  | HR(95% CI) | *P* value |  | HR(95% CI) | *P* value |  | HR(95% CI) | *P* value |  |
| General obesity |  |  |  |  |  |  |  |  |  |
| Overweight (25≤BMI<28, kg/m^2^) | 1.17(1.05-1.32) | 0.0058 |  | 1.11(1.09-1.13) | <0.0001 |  | 1.06(0.94-1.19) | 0.3241 | -- |
| Obesity (BMI≥28kg/m^2^) | 1.55(1.37-1.76) | <0.0001 |  | 1.17(1.13-1.22) | <0.0001 |  | 1.32(1.16-1.51) | <0.0001 | 41.35 |
| Central obesity |  |  |  |  |  |  |  |  |  |
| WC≥90cm in men or ≥85cm in women | 1.48(1.34-1.63) | <0.0001 |  | 1.12(1.10-1.14) | <0.0001 |  | 1.32(1.19-1.46) | <0.0001 | 32.99 |
| WHR≥0.90 in men or ≥0.80 in women | 1.25(1.12-1.38) | <0.0001 |  | 1.10(1.08-1.12) | <0.0001 |  | 1.13(1.02-1.26) | 0.0172 | 45.63 |
| WHTR≥0.60 | 1.26(1.09-1.46) | 0.0018 |  | 1.11(1.09-1.12) | <0.0001 |  | 1.14(0.99-1.32) | 0.0753 | -- |

Abbreviations, BMI, body mass index; CI, confidence interval; HR, hazard ratio; TyG, triglyceride-glucose index; WC, waist circumference; WHR, waist circumference to hip ratio; WHTR, waist circumference to height ratio.

^a^ Decomposition of total associations into natural indirect and natural direct associations was done according to the 2-stage regression method proposed by VanderWeele and performed with the SAS macro provided by ValerWeele. Confidence intervals were calculated according to the delta method procedure.

All models were adjusted for age, sex, education, income, smoking status, drinking status, history of hypertension, diabetes, dyslipidemia, antihypertensive agents, antidiabetic agents, lipide-lowering agents, systolic blood pressure, diastolic blood pressure, total cholesterol, high density lipoprotein cholesterol, and high sensitivity C-reactive protein.

Table S3. Sensitivity analysis on the decomposition of the total association between obesity indexes and the risk of stroke into direct and indirect associations mediated by the TyG index

| Exposures | Association^a^ | | | | | | | | Proportion  mediated, % |
| --- | --- | --- | --- | --- | --- | --- | --- | --- | --- |
|  | Total effect^b^ |  |  | Indirect effect |  |  | Direct effect |  |  |
|  | HR(95% CI) | *P* value |  | HR(95% CI) | *P* value |  | HR(95% CI) | *P* value |  |
| **Excluding participants with baseline FBG ≥7mmol/L** | | | | | | | | | |
| General obesity |  |  |  |  |  |  |  |  |  |
| Overweight (25≤BMI<28, kg/m^2^) | 1.18(1.11-1.26) | <0.0001 |  | 1.05(1.04-1.06) | <0.0001 |  | 1.13(1.06-1.20) | <0.0001 | 30.56 |
| Obesity (BMI≥28kg/m^2^) | 1.43(1.33-1.54) | <0.0001 |  | 1.08(1.06-1.10) | <0.0001 |  | 1.33(1.23-1.43) | <0.0001 | 24.23 |
| Central obesity |  |  |  |  |  |  |  |  |  |
| WC≥90cm in men or ≥85cm in women | 1.30(1.23-1.37) | <0.0001 |  | 1.06(1.04-1.07) | <0.0001 |  | 1.23(1.16-1.30) | <0.0001 | 22.94 |
| WHR≥0.90 in men or ≥0.80 in women | 1.20(1.13-1.27) | <0.0001 |  | 1.05(1.04-1.06) | <0.0001 |  | 1.14(1.08-1.21) | <0.0001 | 27.20 |
| WHTR≥0.60 | 1.32(1.21-1.44) | <0.0001 |  | 1.05(1.04-1.06) | <0.0001 |  | 1.26(1.16-1.37) | <0.0001 | 18.55 |
| **Adding a lag time between exposures and mediator** | | | | | | | | | |
| General obesity |  |  |  |  |  |  |  |  |  |
| Overweight (25≤BMI<28, kg/m^2^) | 1.22(1.14-1.31) | <0.0001 |  | 1.07(1.06-1.08) | <0.0001 |  | 1.14(1.06-1.22) | <0.0001 | 35.48 |
| Obesity (BMI≥28kg/m^2^) | 1.44(1.33-1.56) | <0.0001 |  | 1.12(1.10-1.14) | <0.0001 |  | 1.28(1.18-1.39) | <0.0001 | 35.24 |
| Central obesity |  |  |  |  |  |  |  |  |  |
| WC≥90cm in men or ≥85cm in women | 1.28(1.21-1.36) | <0.0001 |  | 1.07(1.06-1.08) | <0.0001 |  | 1.20(1.13-1.27) | <0.0001 | 30.12 |
| WHR≥0.90 in men or ≥0.80 in women | 1.22(1.14-1.30) | <0.0001 |  | 1.06(1.05-1.07) | <0.0001 |  | 1.15(1.08-1.23) | <0.0001 | 31.55 |
| WHTR≥0.60 | 1.30(1.19-1.43) | <0.0001 |  | 1.06(1.05-1.07) | <0.0001 |  | 1.23(1.12-1.35) | <0.0001 | 24.07 |
| **Adding a lag time between the confounders and set of exposures and mediator** | | | | | | | | | |
| General obesity |  |  |  |  |  |  |  |  |  |
| Overweight (25≤BMI<28, kg/m^2^) | 1.18(1.10-1.26) | <0.0001 |  | 1.06(1.05-1.08) | <0.0001 |  | 1.11(1.04-1.19) | <0.0001 | 39.34 |
| Obesity (BMI≥28kg/m^2^) | 1.36(1.25-1.48) | <0.0001 |  | 1.13(1.10-1.15) | <0.0001 |  | 1.20(1.11-1.31) | <0.0001 | 42.92 |
| Central obesity |  |  |  |  |  |  |  |  |  |
| WC≥90cm in men or ≥85cm in women | 1.28(1.21-1.36) | <0.0001 |  | 1.07(1.05-1.08) | <0.0001 |  | 1.20(1.13-1.28) | <0.0001 | 27.83 |
| WHR≥0.90 in men or ≥0.80 in women | 1.13(1.06-1.20) | <0.0001 |  | 1.04(1.03-1.04) | <0.0001 |  | 1.09(1.02-1.16) | <0.0001 | 31.29 |
| WHTR≥0.60 | 1.41(1.28-1.56) | <0.0001 |  | 1.08(1.06-1.09) | <0.0001 |  | 1.32(1.19-1.45) | <0.0001 | 27.97 |
| **Adding interaction terms into the model** | | | | | | | | | |
| General obesity |  |  |  |  |  |  |  |  |  |
| Overweight (25≤BMI<28, kg/m^2^) | 1.19(1.09-1.29) | <0.0001 |  | 1.07(1.05-1.08) | <0.0001 |  | 1.11(1.02-1.21) | 0.0116 | 26.24 |
| Obesity (BMI≥28kg/m^2^) | 1.42(1.16-1.73) | <0.0001 |  | 1.08(1.04-1.12) | <0.0001 |  | 1.31(1.07-1.61) | 0.0083 | 29.75 |
| Central obesity |  |  |  |  |  |  |  |  |  |
| WC≥90cm in men or ≥85cm in women | 1.31(1.15-1.49) | <0.0001 |  | 1.07(1.06-1.09) | <0.0001 |  | 1.22(1.07-1.39) | 0.003 | 28.51 |
| WHR≥0.90 in men or ≥0.80 in women | 1.24(1.13-1.36) | <0.0001 |  | 1.06(1.05-1.07) | <0.0001 |  | 1.17(1.06-1.28) | 0.0015 | 29.05 |
| WHTR≥0.60 | 1.31(1.21-1.42) | <0.0001 |  | 1.08(1.05-1.10) | <0.0001 |  | 1.22(1.12-1.33) | <0.0001 | 25.83 |

Abbreviations, BMI, body mass index; CI, confidence interval; HR, hazard ratio; TyG, triglyceride-glucose index; WC, waist circumference; WHR, waist circumference to hip ratio; WHTR, waist circumference to height ratio.

^a^ Compared with normal weight participants for general obesity and WC<90cm in men or <85cm in women, WHR<0.90 in men or <0.80 in women, and WHTR<0.60 as a reference for central obesity.

^b^ Decomposition of total associations into natural indirect and natural direct associations was done according to the 2-stage regression method proposed by VanderWeele and performed with the SAS macro provided by ValerWeele. Confidence intervals were calculated according to the delta method procedure.

All models were adjusted for age, sex, education, income, smoking status, drinking status, history of hypertension, diabetes, dyslipidemia, antihypertensive agents, antidiabetic agents, lipide-lowering agents, systolic blood pressure, diastolic blood pressure, total cholesterol, high density lipoprotein cholesterol, and high sensitivity C-reactive protein.

Table S4. Sensitivity analysis on the decomposition of the total association between obesity indexes and the risk of myocardial infarction into direct and indirect associations mediated by the TyG index

| Exposures | Association^a^ | | | | | | | | Proportion  mediated, % |
| --- | --- | --- | --- | --- | --- | --- | --- | --- | --- |
|  | Total effect^b^ |  |  | Indirect effect |  |  | Direct effect |  |  |
|  | HR(95% CI) | *P* value |  | HR(95% CI) | *P* value |  | HR(95% CI) | *P* value |  |
| **Excluding participants with baseline FBG ≥7mmol/L** | | | | | | | | | |
| General obesity |  |  |  |  |  |  |  |  |  |
| Overweight (25≤BMI<28, kg/m^2^) | 1.20(1.06-1.36) | 0.0046 |  | 1.10(1.08-1.13) | <0.0001 |  | 1.09(0.96-1.23) | 0.1927 | -- |
| Obesity (BMI≥28kg/m^2^) | 1.71(1.49-1.96) | <0.0001 |  | 1.15(1.11-1.21) | <0.0001 |  | 1.48(1.28-1.71) | <0.0001 | 32.39 |
| Central obesity |  |  |  |  |  |  |  |  |  |
| WC≥90cm in men or ≥85cm in women | 1.53(1.38-1.71) | <0.0001 |  | 1.11(1.08-1.14) | <0.0001 |  | 1.38(1.24-1.54) | 0.0031 | 28.44 |
| WHR≥0.90 in men or ≥0.80 in women | 1.29(1.16-1.45) | <0.0001 |  | 1.09(1.07-1.11) | <0.0001 |  | 1.18(1.06-1.33) | <0.0001 | 37.13 |
| WHTR≥0.60 | 1.33(1.13-1.56) | 0.0007 |  | 1.09(1.07-1.11) | <0.0001 |  | 1.21(1.03-1.43) | 0.0237 | 34.47 |
| **Adding a lag time between exposures and mediator** | | | | | | | | | |
| General obesity |  |  |  |  |  |  |  |  |  |
| Overweight (25≤BMI<28, kg/m^2^) | 1.24(1.08-1.43) | 0.0022 |  | 1.08(1.06-1.11) | <0.0001 |  | 1.15(1.00-1.32) | 0.0416 | 38.59 |
| Obesity (BMI≥28kg/m^2^) | 1.68(1.44-1.96) | <0.0001 |  | 1.14(1.09-1.19) | <0.0001 |  | 1.48(1.26-1.73) | <0.0001 | 30.10 |
| Central obesity |  |  |  |  |  |  |  |  |  |
| WC≥90cm in men or ≥85cm in women | 1.47(1.30-1.66) | <0.0001 |  | 1.08(1.06-1.11) | <0.0001 |  | 1.36(1.20-1.53) | <0.0001 | 24.07 |
| WHR≥0.90 in men or ≥0.80 in women | 1.22(1.08-1.38) | 0.0015 |  | 1.07(1.06-1.09) | <0.0001 |  | 1.14(1.00-1.29) | 0.0424 | 37.91 |
| WHTR≥0.60 | 1.26(1.05-1.51) | 0.0122 |  | 1.07(1.05-1.09) | <0.0001 |  | 1.18(1.02-1.41) | 0.0156 | 32.70 |
| **Adding a lag time between the confounders and set of exposures and mediator** | | | | | | | | | |
| General obesity |  |  |  |  |  |  |  |  |  |
| Overweight (25≤BMI<28, kg/m^2^) | 1.06(0.93-1.22) | 0.3687 |  | 1.08(1.06-1.10) | <0.0001 |  | 1.00(0.86-1.13) | 0.84715 | -- |
| Obesity (BMI≥28kg/m^2^) | 1.48(1.27-1.74) | <0.0001 |  | 1.16(1.11-1.21) | <0.0001 |  | 1.28(1.09-1.50) | 0.0028 | 42.25 |
| Central obesity |  |  |  |  |  |  |  |  |  |
| WC≥90cm in men or ≥85cm in women | 1.44(1.28-1.62) | <0.0001 |  | 1.08(1.06-1.10) | <0.0001 |  | 1.34(1.19-1.51) | <0.0001 | 27.68 |
| WHR≥0.90 in men or ≥0.80 in women | 1.09(0.96-1.23) | 0.1726 |  | 1.05(1.03-1.06) | <0.0001 |  | 1.04(0.92-1.17) | 0.5211 | -- |
| WHTR≥0.60 | 1.38(1.14-1.68) | 0.0009 |  | 1.09(1.07-1.12) | <0.0001 |  | 1.27(1.04-1.53) | 0.0163 | 30.74 |
| **Adding interaction terms into the model** | | | | | | | | | |
| General obesity |  |  |  |  |  |  |  |  |  |
| Overweight (25≤BMI<28, kg/m^2^) | 1.17(1.04-1.32 | 0.0073 |  | 1.11(1.08-1.14) | <0.0001 |  | 1.06(1.04-1.20) | 0.0334 | 30.69 |
| Obesity (BMI≥28kg/m^2^) | 1.56(1.06-2.30) | 0.0229 |  | 1.12(1.05-1.19) | 0.0004 |  | 1.40(1.05-2.07) | 0.0236 | 32.35 |
| Central obesity |  |  |  |  |  |  |  |  |  |
| WC≥90cm in men or ≥85cm in women | 1.48(1.06-2.08) | 0.0211 |  | 1.10(1.07-1.13) | <0.0001 |  | 1.35(1.06-1.89) | 0.0188 | 31.49 |
| WHR≥0.90 in men or ≥0.80 in women | 1.25(1.05-1.48) | 0.0110 |  | 1.09(1.07-1.12) | <0.0001 |  | 1.14(1.06-1.36) | 0.0321 | 23.34 |
| WHTR≥0.60 | 1.27(0.99-1.62) | 0.057 |  | 1.09(1.04-1.14) | 0.0004 |  | 1.17(1.01-1.50) | 0.0226 | 22.57 |

Abbreviations, BMI, body mass index; CI, confidence interval; HR, hazard ratio; TyG, triglyceride-glucose index; WC, waist circumference; WHR, waist circumference to hip ratio; WHTR, waist circumference to height ratio.

^a^ Compared with normal weight participants for general obesity and WC<90cm in men or <85cm in women, WHR<0.90 in men or <0.80 in women, and WHTR<0.60 as a reference for central obesity.

^b^ Decomposition of total associations into natural indirect and natural direct associations was done according to the 2-stage regression method proposed by VanderWeele and performed with the SAS macro provided by ValerWeele. Confidence intervals were calculated according to the delta method procedure.

All models were adjusted for age, sex, education, income, smoking status, drinking status, history of hypertension, diabetes, dyslipidemia, antihypertensive agents, antidiabetic agents, lipide-lowering agents, systolic blood pressure, diastolic blood pressure, total cholesterol, high density lipoprotein cholesterol, and high sensitivity C-reactive protein.

Table S5. Percentage excess risk mediated by the TyG index

| Parameters | Confounder adjusted HR | Confounder and the TyG index adjusted HR | Excess risk (%) mediated by the TyG index |
| --- | --- | --- | --- |
| Normal weight | Reference | Reference | -- |
| General obesity |  |  |  |
| Overweight (25≤BMI<28, kg/m^2^) | 1.18(1.12-1.24) | 1.09(1.04-1.15) | 44.84 |
| Obesity (BMI≥28kg/m^2^) | 1.44(1.36-1.53) | 1.28(1.20-1.36) | 32.54 |
| Central obesity |  |  |  |
| WC≥90cm in men or ≥85cm in women | 1.34(1.28-1.41) | 1.24(1.18-1.30) | 28.04 |
| WHR≥0.90 in men or ≥0.80 in women | 1.24(1.18-1.30) | 1.16(1.10-1.22) | 30.44 |
| WHTR≥0.60 | 1.30(1.22-1.39) | 1.21(1.13-1.30) | 27.49 |

Abbreviations, BMI, body mass index; CI, confidence interval; HR, hazard ratio; TyG, triglyceride-glucose index; WC, waist circumference; WHR, waist circumference to hip ratio; WHTR, waist circumference to height ratio.

All models were adjusted for age, sex, education, income, smoking status, drinking status, history of hypertension, diabetes, dyslipidemia, antihypertensive agents, antidiabetic agents, lipide-lowering agents, systolic blood pressure, diastolic blood pressure, total cholesterol, high density lipoprotein cholesterol, and high sensitivity C-reactive protein.

Table S6. Decomposition of the total association between obesity indexes and the risk of CVD into direct and indirect associations mediated by the TyG index stratified by age

| Exposures | Age | Association^a^ | | | | | | | | | Proportion  mediated, % |
| --- | --- | --- | --- | --- | --- | --- | --- | --- | --- | --- | --- |
|  |  | Total effect^b^ |  |  | Indirect effect |  |  | Direct effect | |  |  |
|  |  | HR(95% CI) | *P* value |  | HR(95% CI) | *P* value |  | HR(95% CI) | | *P* value |  |
| General obesity |  |  |  |  |  |  |  |  | |  |  |
| Overweight (25≤BMI<28kg/m^2^) | <60 years | 1.25(1.17-1.34) | <0.0001 |  | 1.09(1.07-1.10) | <0.0001 |  | 1.15(1.08-1.24) | | <0.0001 | 39.28 |
|  | ≥60 years | 1.07(0.99-1.17) | 0.1000 |  | 1.05(1.04-1.06) | <0.0001 |  | 1.02(0.94-1.11) | | 0.6320 | -- |
| Obesity (BMI≥28kg/m^2^) | <60 years | 1.53(1.42-1.65) | <0.0001 |  | 1.14(1.12-1.17) | <0.0001 |  | 1.34(1.24-1.45) | | <0.0001 | 35.83 |
|  | ≥60 years | 1.23(1.11-1.35) | <0.0001 |  | 1.08(1.06-1.11) | <0.0001 |  | 1.13(1.02-1.25) | | <0.0001 | 41.71 |
| Central obesity |  |  |  |  |  |  |  |  | |  |  |
| WC≥90cm in men or ≥85cm in women | <60 years | 1.38(1.30-1.47) | <0.0001 |  | 1.09(1.08-1.11) | <0.0001 |  | 1.27(1.19-1.34) | | <0.0001 | 30.25 |
|  | ≥60 years | 1.22(1.13-1.32) | <0.0001 |  | 1.07(1.05-1.08) | <0.0001 |  | 1.22(1.13-1.32) | | 0.0005 | 34.01 |
| WHR≥0.90 in men or ≥0.80 in women | <60 years | 1.29(1.22-1.38) | 0.0013 |  | 1.08(1.07-1.09) | <0.0001 |  | 1.20(1.13-1.28) | | 0.0332 | 31.03 |
|  | ≥60 years | 1.13(1.05-1.22) | 0.0016 |  | 1.05(1.04-1.06) | <0.0001 |  | 1.08(1.00-1.17) | | 0.00051 | 39.01 |
| WHTR≥0.60 | <60 years | 1.44(1.31-1.58) | <0.0001 |  | 1.08(1.07-1.10) | <0.0001 |  | 1.33(1.21-1.46) | | <0.0001 | 25.26 |
|  | ≥60 years | 1.17(1.06-1.29) | 0.0023 |  | 1.06(1.04-1.07) | <0.0001 |  | | 1.10(1.00-1.22) | 0.0456 | 29.37 |

Abbreviations, BMI, body mass index; CI, confidence interval; CVD, cardiovascular disease; HR, hazard ratio; TyG, triglyceride-glucose index; WC, waist circumference; WHR, waist circumference to hip ratio; WHTR, waist circumference to height ratio.

^a^ Compared with normal weight participants for general obesity and WC<90cm in men or <85cm in women, WHR<0.90 in men or <0.80 in women, and WHTR<0.60 as a reference for central obesity.

^b^ Decomposition of total associations into natural indirect and natural direct associations was done according to the 2-stage regression method proposed by VanderWeele and performed with the SAS macro provided by ValerWeele. Confidence intervals were calculated according to the delta method procedure.

All models were adjusted for age, education, income, smoking status, and drinking status, history of hypertension, diabetes, dyslipidemia, antihypertensive agents, antidiabetic agents, lipide-lowering agents, systolic blood pressure, diastolic blood pressure, total cholesterol, high density lipoprotein cholesterol, and high sensitivity C-reactive protein.

Table S7. Decomposition of the total association between obesity indexes and the risk of CVD into direct and indirect associations mediated by the TyG index stratified by sex

| Exposures | Sex | Association^a^ | | | | | | | | Proportion  mediated, % |
| --- | --- | --- | --- | --- | --- | --- | --- | --- | --- | --- |
|  |  | Total effect^b^ |  |  | Indirect effect |  |  | Direct effect |  |  |
|  |  | HR(95% CI) | *P* value |  | HR(95% CI) | *P* value |  | HR(95% CI) | *P* value |  |
| General obesity |  |  |  |  |  |  |  |  |  |  |
| Overweight (25≤BMI<28kg/m^2^) | Women | 1.21(1.03-1.43) | 0.0220 |  | 1.08(1.06-1.11) | <0.0001 |  | 1.12(0.95-1.32) | 0.1789 | -- |
|  | Men | 1.17(1.11-1.24) | <0.0001 |  | 1.08(1.06-1.09) | <0.0001 |  | 1.09(1.03-1.15) | 0.0023 | 47.33 |
| Obesity (BMI≥28kg/m^2^) | Women | 1.63(1.37-1.94) | <0.0001 |  | 1.12(1.07-1.17) | <0.0001 |  | 1.46(1.22-1.74) | <0.0001 | 27.17 |
|  | Men | 1.41(1.33-1.51) | <0.0001 |  | 1.13(1.11-1.15) | <0.0001 |  | 1.25(1.17-1.34) | <0.0001 | 38.64 |
| Central obesity |  |  |  |  |  |  |  |  |  |  |
| WC≥90cm in men or ≥85cm in women | Women | 1.41(1.21-1.63) | <0.0001 |  | 1.09(1.07-1.12) | <0.0001 |  | 1.29(1.11-1.49) | 0.0009 | 29.72 |
|  | Men | 1.33(1.27-1.40) | <0.0001 |  | 1.08(1.07-1.10) | <0.0001 |  | 1.23(1.17-1.29) | <0.0001 | 30.95 |
| WHR≥0.90 in men or ≥0.80 in women | Women | 1.41(1.11-1.78) | 0.0046 |  | 1.10(1.07-1.13) | <0.0001 |  | 1.28(1.01-1.63) | 0.0419 | 31.16 |
|  | Men | 1.23(1.17-1.29) | <0.0001 |  | 1.07(1.06-1.07) | <0.0001 |  | 1.16(1.10-1.21) | <0.0001 | 32.69 |
| WHTR≥0.60 | Women | 1.44(1.22-1.69) | <0.0001 |  | 1.07(1.05-1.09) | <0.0001 |  | 1.35(1.14-1.58) | 0.0003 | 20.67 |
|  | Men | 1.26(1.17-1.36) | <0.0001 |  | 1.07(1.06-1.09) | <0.0001 |  | 1.18(1.09-1.27) | <0.0001 | 33.35 |

Abbreviations, BMI, body mass index; CI, confidence interval; CVD, cardiovascular disease; HR, hazard ratio; TyG, triglyceride-glucose index; WC, waist circumference; WHR, waist circumference to hip ratio; WHTR, waist circumference to height ratio.

^a^ Compared with normal weight participants for general obesity and WC<90cm in men or <85cm in women, WHR<0.90 in men or <0.80 in women, and WHTR<0.60 as a reference for central obesity.

^b^ Decomposition of total associations into natural indirect and natural direct associations was done according to the 2-stage regression method proposed by VanderWeele and performed with the SAS macro provided by ValerWeele. Confidence intervals were calculated according to the delta method procedure.

All models were adjusted for age, sex, education, income, smoking status, drinking status, history of hypertension, diabetes, dyslipidemia, antihypertensive agents, antidiabetic agents, lipide-lowering agents, systolic blood pressure, diastolic blood pressure, total cholesterol, high density lipoprotein cholesterol, and high sensitivity C-reactive protein.


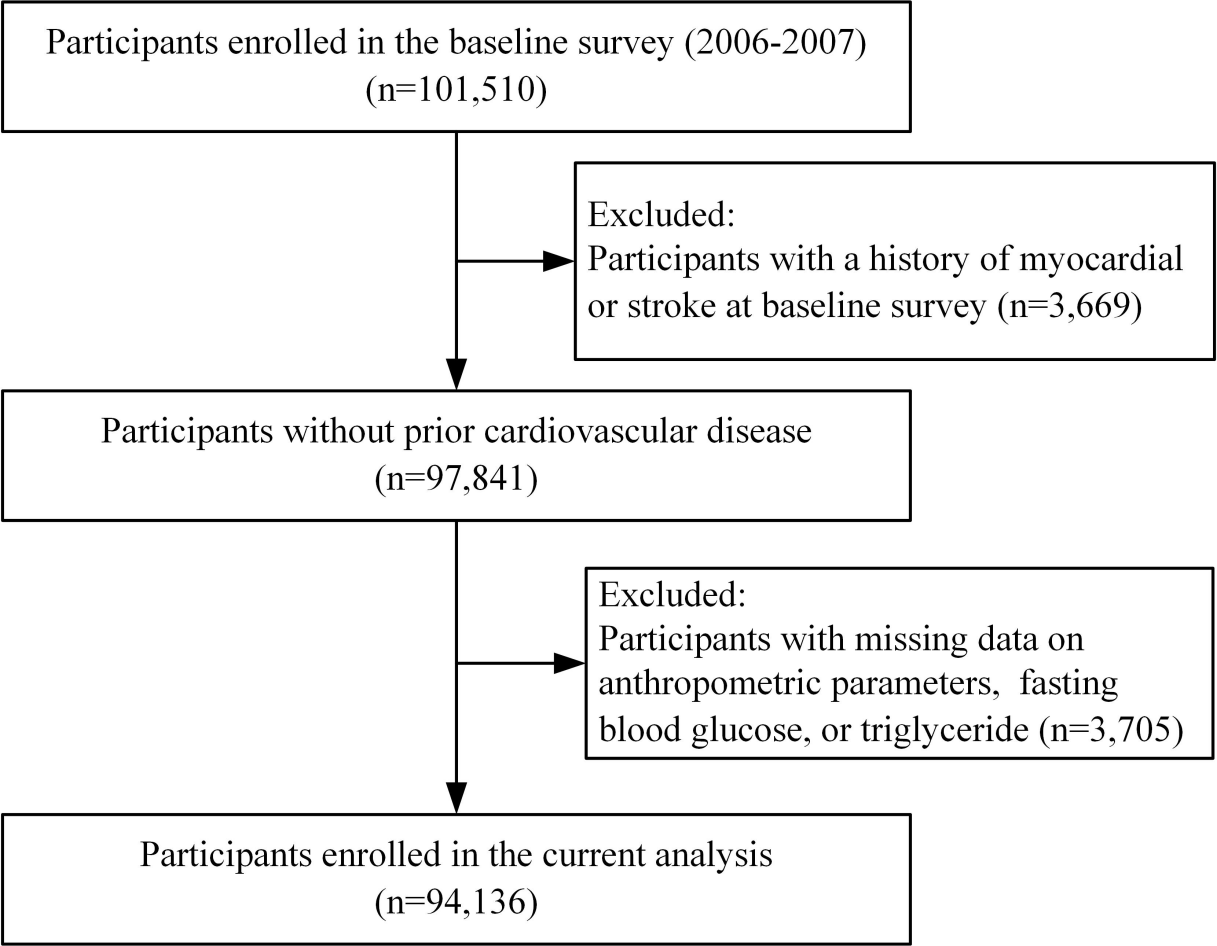


Figure S1. Flowchart of the study


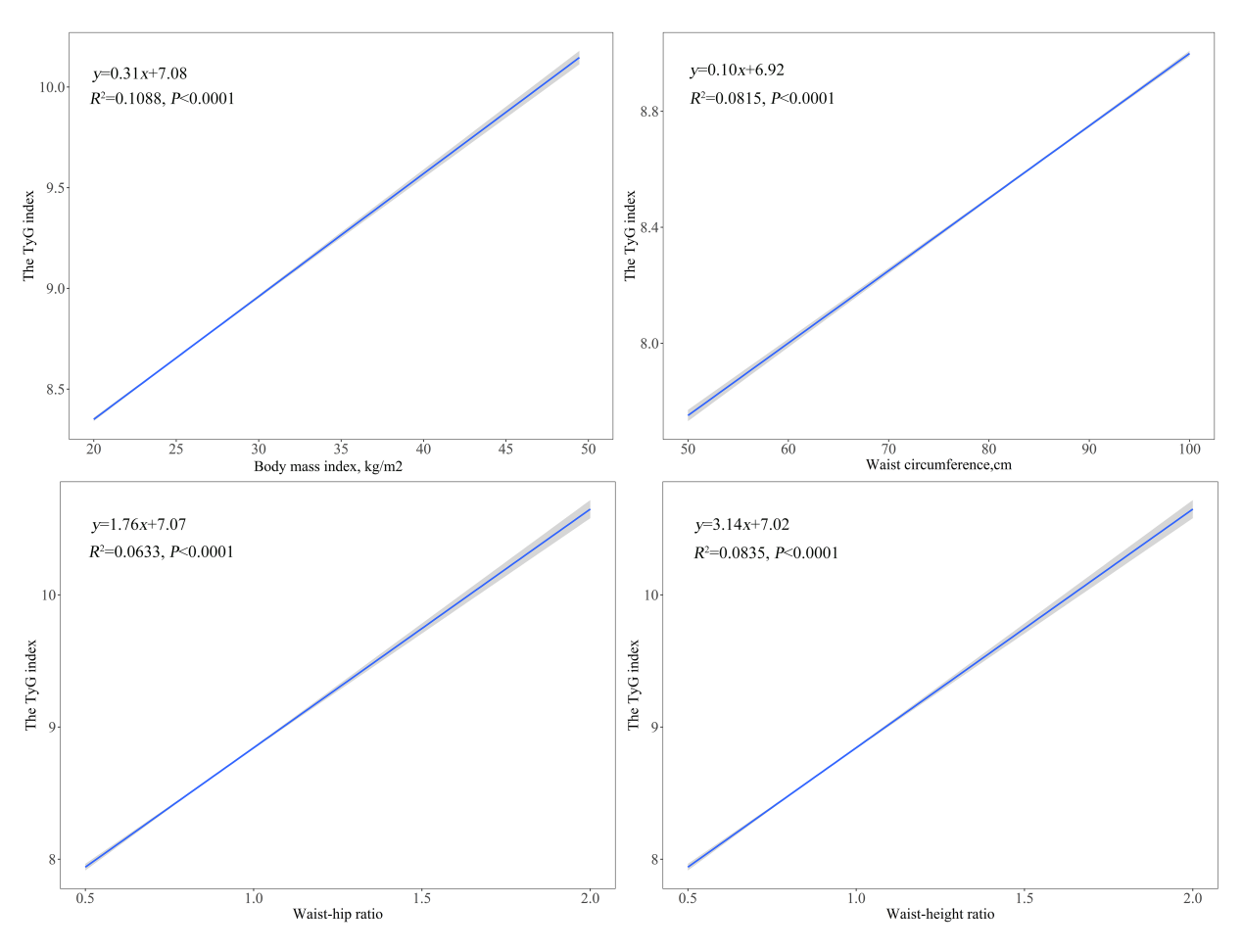


Figure S2. Correlation between obesity index and the TyG index

Abbreviations: TyG index, triglyceride-glucose index.

All models were adjusted for age, sex, education, income, smoking status, drinking status, history of hypertension, diabetes, dyslipidemia, antihypertensive agents, antidiabetic agents, lipide-lowering agents, systolic blood pressure, diastolic blood pressure, total cholesterol, and high sensitivity C-reactive protein.
